# Asymmetric ^18^F-fluorination for applications in positron emission tomography

**DOI:** 10.1039/c5sc04229a

**Published:** 2015-12-17

**Authors:** Faye Buckingham, Véronique Gouverneur

**Affiliations:** a University of Oxford , Chemistry Research Laboratory , 12 Mansfield Road , OX1 3UQ , Oxford , UK . Email: veronique.gouverneur@chem.ox.ac.uk

## Abstract

Positron emission tomography (PET) is becoming more frequently used by medicinal chemists to facilitate the selection of the most promising lead compounds for further evaluation. For PET, this entails the preparation of ^11^C- or ^18^F-labeled drugs or radioligands. With the importance of chirality and fluorine substitution in drug development, chemists can be faced with the challenge of preparing enantiopure molecules featuring the ^18^F-tag on a stereogenic carbon. Asymmetric ^18^F-fluorination is an emerging field of research that provides an alternative to resolution or conventional S_N_2-based radiochemistry. To date, both transition metal complexes and organomediators have been successfully employed for ^18^F-incorporation at a stereogenic carbon.

## Abstract 2

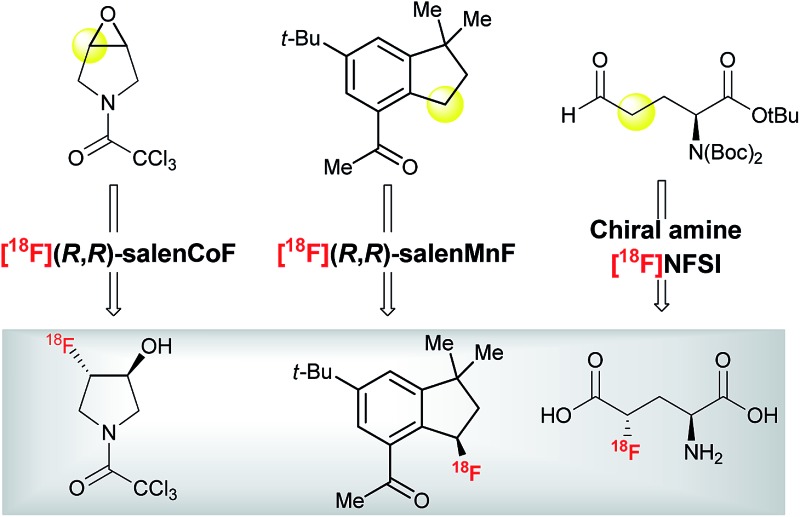
To date, both transition metal complexes and organomediators have been employed for enantiocontrolled ^18^F-incorporation as an alternative to conventional resolution of S_N_2-based radiochemistry.

## 1. Introduction

It is universally recognized that molecular chirality has a direct impact on function. In nature, many essential biological molecules exist only in one of two possible mirror-image structures, either because they possess a chiral unit or through their overall structure.^1^ In the context of drug development, chirality dominates and it has been accepted since the early 1980s that most of the biological activity observed for a racemate often resides within a single enantiomer.^2^ As a result, it was anticipated that the proportion of racemic new molecular entities (NMEs) would decrease over time and possibly vanish. A recent survey indicates that the number of enantiopure NMEs approved since the mid-1990s has indeed increased, but the development and approval of racemic compounds remains a viable approach.^3^ The deeply rooted importance of chirality in the pharmaceutical industry^4^ and other areas, such as material science, has encouraged much research in asymmetric synthesis and catalysis, two active fields of modern chemistry.^5^

**Fig. 1:**
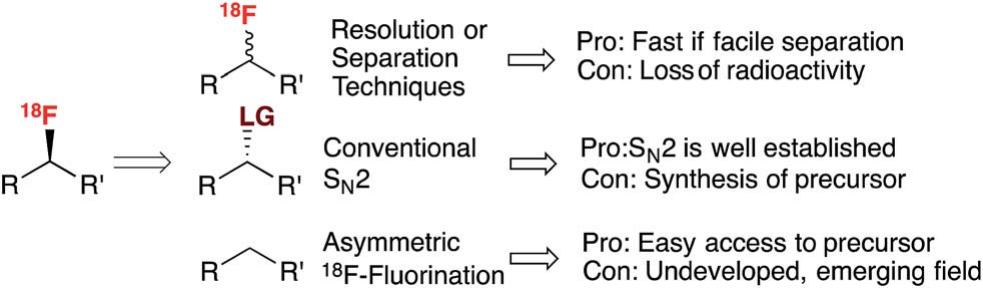
^18^F–C stereogenicity: resolution or separation techniques, conventional S_N_2, and asymmetric ^18^F-fluorination.

The scientific complexity of drug discovery and the commercial challenges currently facing the pharmaceutical industry have led medicinal chemists to consider Positron Emission Tomography (PET)^6^ more frequently as a technology for the identification of the most promising lead compounds much earlier in the drug discovery pipeline.^7^ PET is a non-invasive quantitative imaging modality that can be employed to study drug pharmacokinetics and pharmacodynamics, and the relationship of these pharmacological characteristics to the behavioral, therapeutic and toxic properties of drugs. With the current trend in the pharmaceutical industry to develop optically pure products, PET can also assess the behavior of individual enantiomers in living systems.^8^ For chiral radio-pharmaceuticals used in the clinic, the administration of a single enantiomer is beneficial in terms of minimizing amount of radioactivity for the patient and, in some cases, by reducing background uptake due to non-specific retention of the inactive enantiomer. The advantageous characteristics of the positron emitting isotope ^18^F,^9^ and the prominent position of fluorine substitution in drug discovery^10^ have fuelled an upsurge of interest in ^18^F-radiochemistry, with the appearance of novel methods for ^18^F-labeling inspired by modern ^19^F chemistry. Despite these advances, the production of chiral non-racemic ^18^F-labeled drugs remains challenging, especially when the ^18^F-tag is located on a stereogenic carbon. Fig. 1 presents the various approaches one may consider for this latter scenario.

Radiochemists would typically consider the separation of ^18^F-labeled stereoisomers using High Performance Liquid Chromatography (HPLC) in preference to overcoming the obstacles associated with stereoselective or asymmetric ^18^F-fluorination; this is despite the fact that such separation leads to a substantial loss of radioactivity (50% loss for the separation of two enantiomers). In this essay, we discuss the challenges associated with ^18^F-incorporation onto a stereogenic carbon and the current state of play of this field of research; in the conclusive remarks, we question how important such developments are for drug developers and PET radiochemists.

## 2. ^18^F–C bond formation and stereogenicity

The slow progress of ^18^F-radiochemistry in comparison with ^19^F-chemistry is commensurate with the various hurdles associated with ^18^F-labeling. Most academic and clinical research laboratories are not equipped to handle the cyclotron produced radioisotope ^18^F, a non-trivial limitation preventing fast development in ^18^F-radiochemistry. The half-life of the positron emitter ^18^F (109 min) imposes time constraints that are not compatible with the lengthy reaction time required for many late stage ^19^F-fluorinations, and the stoichiometry of ^18^F-radiochemical processes may lead to significant differences in terms of reaction kinetics, in addition to complications for purification.^9^ The ^18^F source is indeed employed in nano- or picomolar quantity and is therefore in large sub-stoichiometry with respect to the precursor. Furthermore, for radiopharmaceuticals, additional complications may arise during isolation and formulation due to radiolytic decomposition.^11^ Another significant difference between ^18^F and ^19^F chemistries stems from the preference for reactions using [^18^F]fluoride instead of [^18^F]F_2_. [^18^F]Fluoride is easier to produce and to handle than [^18^F]F_2_ and is therefore widely available. Importantly, use of a nucleophilic ^18^F source leads to ^18^F-labeled molecules in higher specific activity, an advantageous property that widens considerably the range of PET studies possible to support drug discovery programs as well as clinical studies. Despite the high strength of the C–F bond (for CH_3_F, BDE = 109.9 kcal mol^–1^),^12^ metabolic paths leading to radiodefluorination with release of [^18^F]fluoride are problematic for tracers formulated with high specific activity and concentration, because [^18^F]fluoride binds strongly to the skeletal system. This effect is minimized with ^18^F-labeled aryl fluorides, which are more stable towards defluorination than alkyl fluoride. The intrinsic sp^3^ hybridization of stereogenic carbons makes chiral ^18^F-labeled tracers susceptible to rapid metabolic degradation through oxidation and/or elimination pathways. Some reports suggest that the use of ^18^F-labeled cycloalkyl fluoride, and more generally ^18^F-incorporation onto secondary instead of primary carbon atoms, typically enhances metabolic resistance;^13^ these structurally refined compounds may feature ^18^F–C stereogenicity and pose additional radiosynthetic challenges.

### 2.1 The conventional S_N_2 approach

**Scheme 1:**
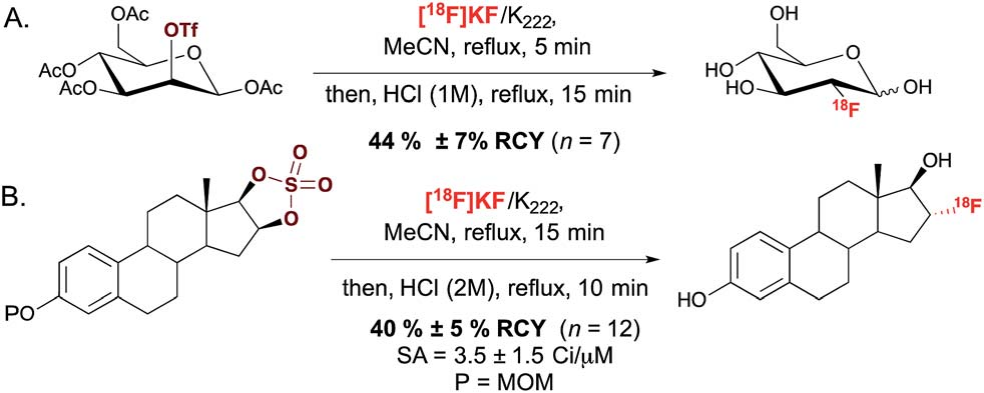
(A.) Radiosynthesis of 2-[^18^F]fluoro-2-deoxy-d-glucose ([^18^F]FDG). (B.) Radiosynthesis of 16-α-[^18^F]fluoroestradiol ([^18^F]FES). SA = specific activity. MOM = methoxymethyl.

The most commonly employed radiotracer in the clinic is 2-[^18^F]fluoro-2-deoxy-d-glucose ([^18^F]FDG), a non-racemic chiral molecule which presents the ^18^F-substituent itself on a stereogenic carbon.^14^ Its preparation, and more generally ^18^F-fluorination at a stereogenic carbon, involves S_N_2 displacement of a leaving group positioned on an enantiomerically pure precursor (Scheme 1A). The leaving groups typically selected for displacement are triflate, tosylate or halide functionalities. Alternatively, one could consider the regio- and stereoselective ring opening of a cyclic sulfate, a reaction that was successfully applied for the radiosynthesis of 16-α-[^18^F]fluoroestradiol ([^18^F]FES) (Scheme 1B).^15^

**Scheme 2:**
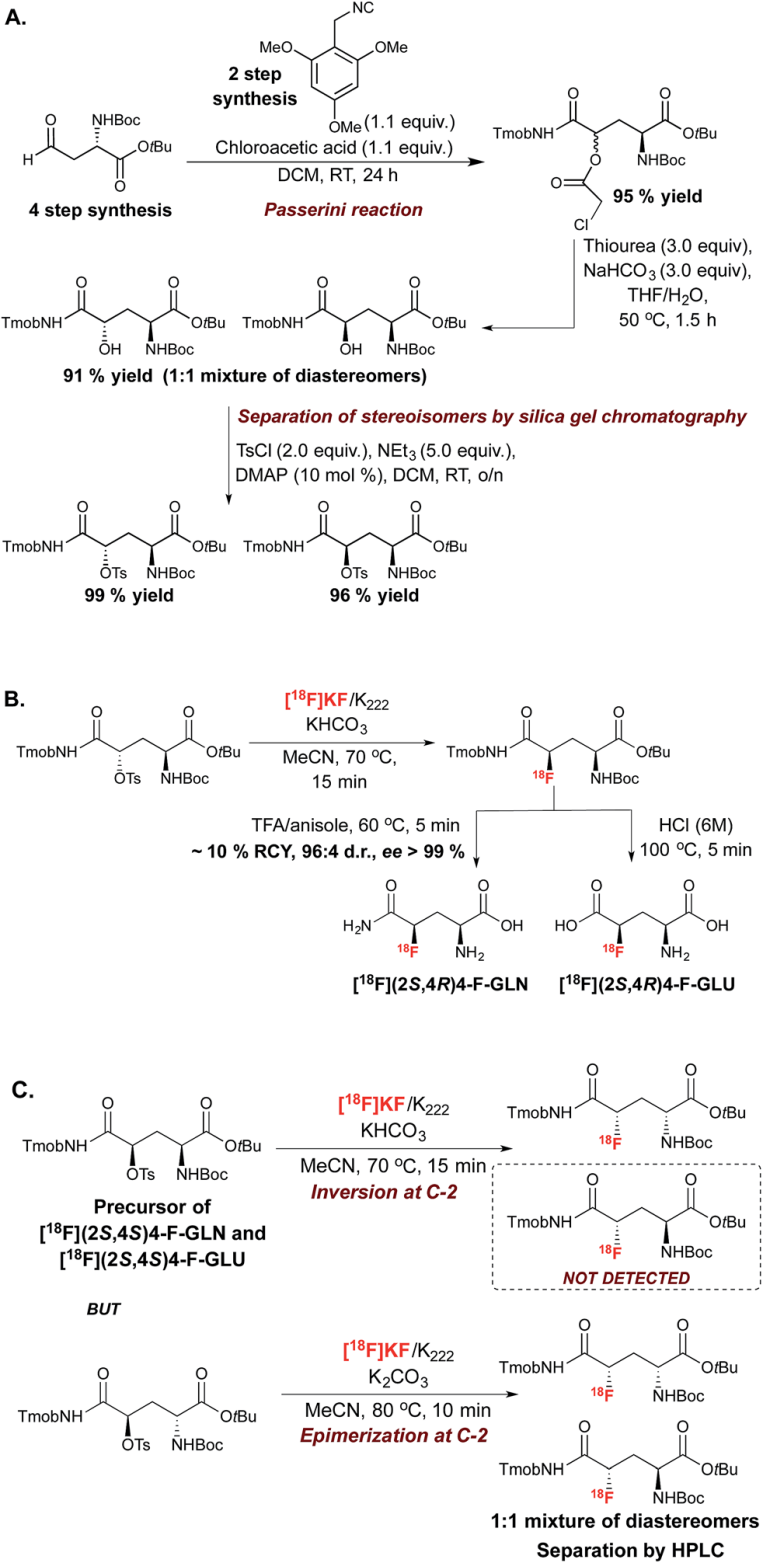
(A.) Synthesis of the substrates for the radiosynthesis of [^18^F]4 F-GLU and [^18^F]4-F-GLN. (B.) Radiosynthesis of [^18^F](2*S*,4*R*)4-F-GLU and [^18^F](2*S*,4*R*)4-F-GLN. (C.) Radiosynthesis of [^18^F](2*S*,4*S*)4-F-GLU and [^18^F](2*S*,4*S*)4-F-GLN precursors. Tmob = 2,4,6-trimethoxybenzyl.

This S_N_2-based approach typically employs high temperatures and is limited to substrates that are not prone to decomposition under the reaction conditions. Also, since fluoride is a potent base as well as a nucleophile, both the substrates and newly formed ^18^F-labeled product should be resistant to elimination and racemization (or epimerization) under the ^18^F-fluorination conditions. The possibility of incomplete inversion and the time-consuming synthesis of enantiopure precursors are further complications associated with this conventional S_N_2 strategy. Such challenges are possibly best illustrated with the synthesis of ^18^F-labeled 4-fluoro-l-glutamine (4F-GLN) and 4-fluoro-l-glutamic acid (4F-GLU) (Scheme 2).^16^

Protected precursors for the two possible diastereomers for these radiotracers were prepared by nucleophilic displacement with [^18^F]fluoride of the tosyl leaving group installed onto the requisite protected substrates. The challenges imposed by this strategy include the multi-step synthesis and fragile stability of the substrates, the occurrence of stereochemical erosion at C-2, and competitive cyclization upon ^18^F-fluorination. *In vitro* and *in vivo* studies were conducted with ^18^F-(2*S*,4*R*)4F-GLN and ^18^F-(2*S*,4*R*)4F-GLU, which are easier to access and to purify than diastereomers ^18^F-(2*S*,4*S*)4F-GLN and ^18^F-(2*S*,4*S*)4F-GLU. The development of these demanding ^18^F-labeling experiments was however worthwhile, as evaluation studies showed that tumor cell uptake of ^18^F-(2*S*,4*R*)4F-GLN is higher than that of ^18^F-(2*S*,4*R*)4F-GLU, likely due to increased amino acid transport activity, protein incorporation, and non-protein metabolic pathways such as glutaminolysis. In contrast, ^18^F-(2*S*,4*R*)4F-GLU is not incorporated into protein, with the uptake believed to be controlled by the transporter. *In vivo* studies showed that although ^18^F-(2*S*,4*R*)4F-GLN exhibited a higher uptake and longer retention in rats bearing 9L tumor xenographs, ^18^F-(2*S*,4*R*)4F-GLU showed a slightly higher tumor-to-background ratio due to a faster background clearance. Both ^18^F-(2*S*,4*R*)4F-GLN and ^18^F-(2*S*,4*R*)4F-GLU are useful as tumor metabolic imaging agents.^17^

### 2.2. Asymmetric ^18^F-fluorination with metals

More recent reports have sidestepped the challenges associated with stereoselective S_N_2 substitution, by favouring an alternative approach in which the product stereochemistry is set by an enantioselective ^18^F-fluorination. In 2011, the demonstration that a transition metal allowed for regioselective allylic ^18^F-incorporation opened numerous opportunities towards stereoselective or asymmetric ^18^F-fluorination directly inspired by research carried out with the non-radioactive isotope ^19^F.^18^ Allyl carbonates were found to react with the [^18^F]fluoride source [^18^F]TBAF in the presence of Pd(dba)_2_^19^ or [Ir(COD)Cl]_2,_^20^ leading to branched, linear *E* or linear *Z* ^18^F-labeled allyl fluorides with clean control over product selectivity. The demonstration that metal mediated ^18^F–Csp^3^ bond formation is possible, boded well for the use of these and other transition metals for stereocontrolled ^18^F-fluorination.

#### Cobalt mediated hydrofluorination of epoxides

**Scheme 3:**
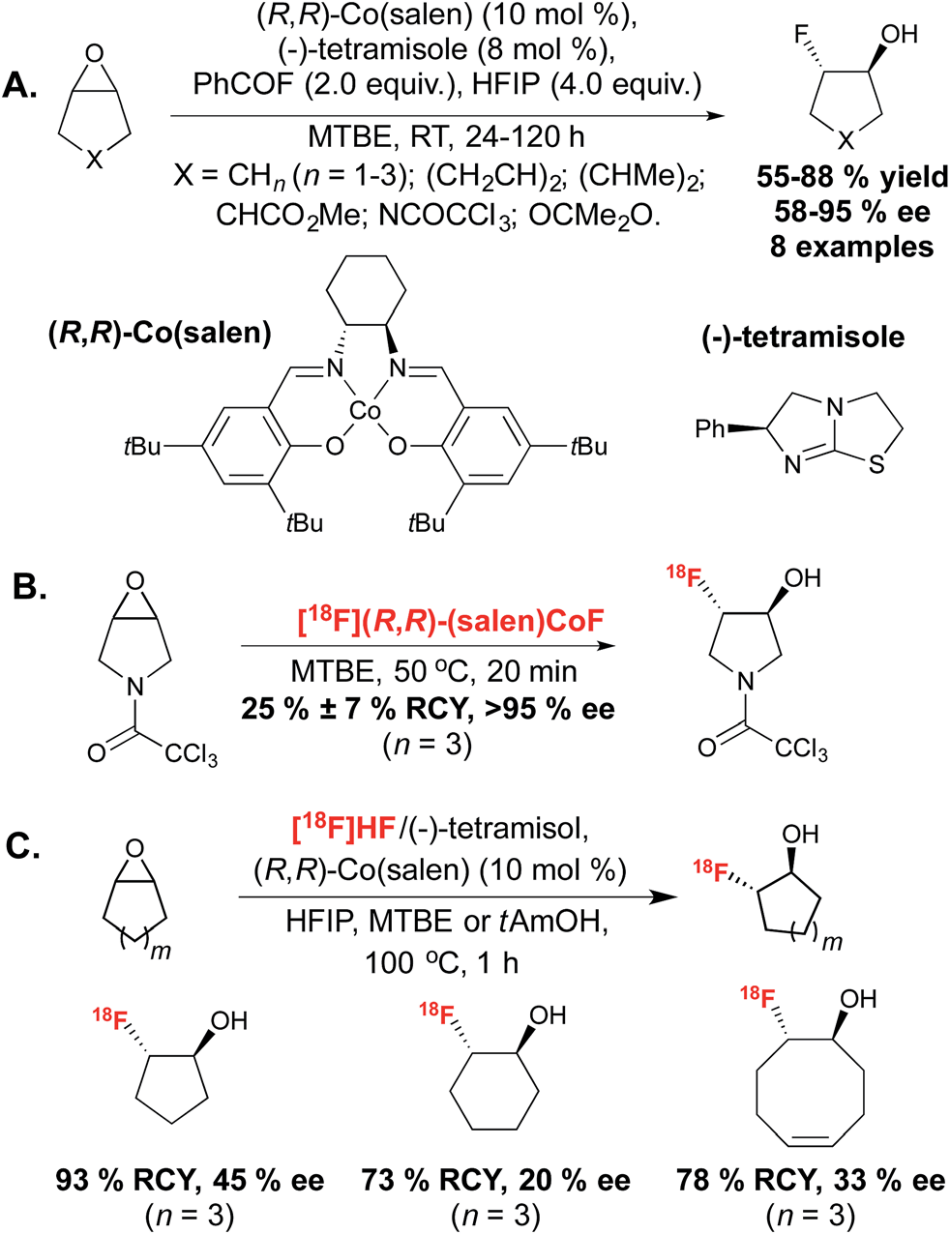
(A.) Enantioselective opening of *meso* epoxides by fluoride catalysed by (*R*,*R*)-Co(salen) and the chiral amine (–)-tetramisole. (B.) Radiosynthesis of an enantiopure fluorohydrin with [^18^F](*R*,*R*)-Co(salen). (C.) Enantioselective desymmetrization of *meso* epoxides with [^18^F]HF and (*R*,*R*)-Co(salen).

Building on the seminal work of Bruns and Haufe in 2000,^21^ Doyle reported the enantioselective ring opening of *meso* and terminal epoxides by fluoride, catalysed by (*R*,*R*)-Co(salen) and the chiral amine (–)-tetramisole (Scheme 3A).^22^

Whilst the previous work had identified that chiral Lewis acid mediated epoxide ring-opening reactions with HF·pyridine complex suffered from low enantioselectivity due to racemic background reaction and catalyst degradation, Doyle discovered that a combination of benzoyl fluoride and hexafluoroisopropanol (HFIP) provided mild release of fluoride, thereby enabling the formation of the fluorohydrin product with excellent enantiocontrol. The reaction likely progressed *via* amine catalyzed *in situ* slow formation of HF, which in turn could generate the active (salen)Co(iii) fluorine complex. The desymmetrization of a range of *meso* epoxides occurred with enantiomeric excesses (ee) reaching up to 95%. In a later report, more detailed mechanistic studies on this reaction led to an improved protocol with low catalyst loading.^23^ In 2014, Doyle in collaboration with Kung described extension of this methodology to ^18^F-radiofluorination.^24^ The [^18^F](salen)CoF complex was synthesized by reaction of (*R*,*R*)-(salen)CoOTs with [^18^F]fluoride eluted from an ion-exchange cartridge, without the requirement for azeotropic drying, and was employed for reaction with epoxide precursors in methyl-*tert*-butylether (MTBE) to achieve hydrofluorination with high RCY within 20 minutes. An excellent level of enantiocontrol was demonstrated, despite an increase of reaction temperature to 50 °C. The synthesis of a range of radiotracers was well tolerated. In this report, the majority of substrates were terminal epoxides and only one example in which desymmetrization of a *meso* epoxide led to a product with a ^18^F-substituted carbon stereocenter was disclosed (Scheme 3B). In 2013, Revunov and Zhuravlev took inspiration from the cobalt mediated enantioselective epoxide opening reaction.^25^ This work employed [^18^F]fluoride treated with H_2_SO_4_, proposed by the authors to form [^18^F]HF, which could be trapped by addition of (–)-tetramisole. The reaction took place by addition of (*R*,*R*)-Co(salen), HFIP and an epoxide precursor in either MTBE or 2-methyl-2-butanol (*t*AmOH) as solvent (Scheme 3C). The use of HFIP was found to be crucial to product formation. The reaction was performed at 100 °C for 1 hour and afforded 3 examples of [^18^F]fluorohydrin products from cyclic epoxides with good RCY but low ee values, which the authors hypothesized was due to the high reaction temperature.

#### Manganese mediated benzylic C–H activation

A report by Groves and co-workers in 2014 set out to avoid a pre-functionalized approach to ^18^F-labeling with the development of a method which involved the direct replacement of a benzylic sp^3^ hydrogen with fluorine.^26^

**Scheme 4:**
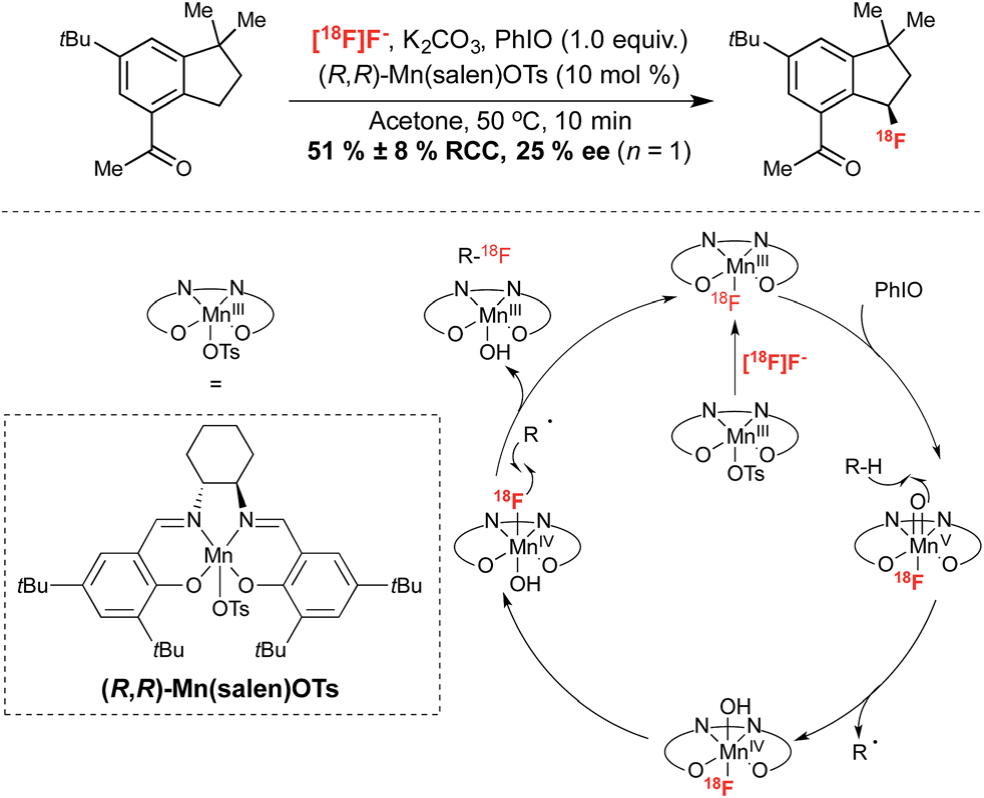
Enantioselective benzylic ^18^F-fluorination with [^18^F]fluoride through C–H functionalization.

This elegant transformation installs fluorine substitution onto a stereogenic carbon. In an extension of the ^19^F-fluorination reactions reported by the same group,^27^ this procedure facilitated the ^18^F-fluorination of a wide range of precursors containing benzylic C–H bonds. Analogously to the cobalt mediated reaction of Doyle, the reaction was proposed to proceed *via* formation of a [^18^F](salen)Mn fluorine complex, generated by reaction of Mn(salen)OTs with [^18^F]fluoride from an ion-exchange cartridge. In this case, this complex was proposed to undergo oxidation in the presence of iodosobenzene. Reaction at 50 °C in acetone for 10 minutes afforded ^18^F-labeled products with RCY reaching up to 72%, with an array of functional groups tolerated under these conditions. Starting with 3.5 mCi of [^18^F]fluoride, ^18^F-labeled celestolide was obtained in 10% non-decay corrected RCY with a specific activity of 2.68 Ci μmol^–1^ (end of bombardment). In a singular example of the potential for synthesis of enantioenriched products employing this protocol, the Mn(salen)OTs mediated ^18^F-fluorination of celestolide afforded the radiolabeled product with an ee of 25% (Scheme 4). Currently the optimization of this reaction in terms of ee has yet to be reported. Nevertheless, this preliminary example highlights the potential opportunity for asymmetric ^18^F-fluorination with this methodology, which advantageously utilizes nucleophilic [^18^F]fluoride and employs precursors which do not require pre-functionalization with a leaving group.

### 2.3. Organomediated asymmetric ^18^F-fluorination

**Scheme 5:**
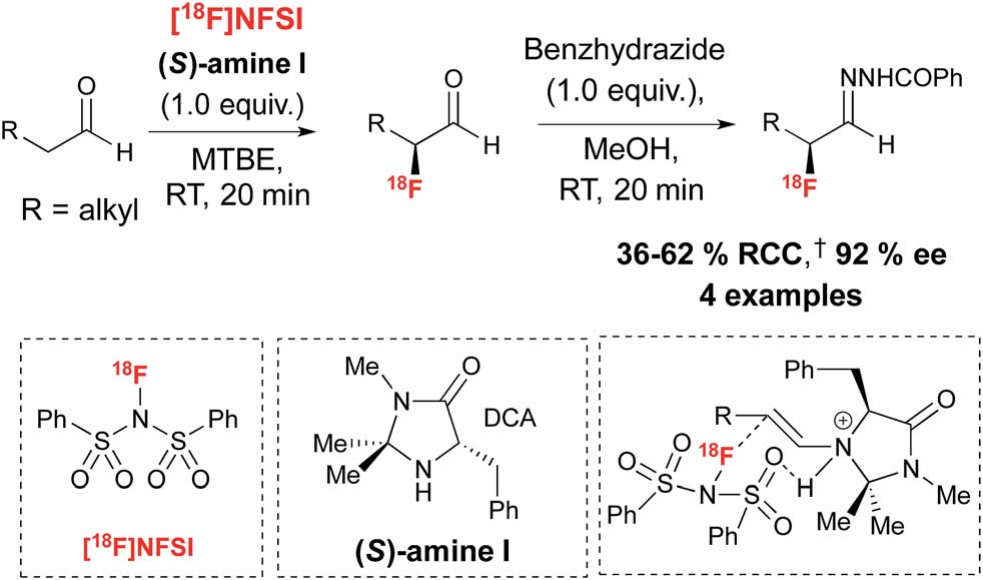
Chiral imidazolidinone (*S*)-I for the enantioselective ^18^F-fluorination of aldehydes with [^18^F]NFSI. ^†^RCC determined by radio-HPLC relative to [^18^F]NFSI. DCA = dichloroacetic acid. For [^18^F]NFSI, SA = 0.05 Ci μmol^–1^, (*n* = 3).

Encouraged by the benefits associated with organocatalysis,^28^ our research group recently reported a metal-free approach^29^ to asymmetric ^18^F-fluorination by taking inspiration from the wealth of literature surrounding organocatalyzed asymmetric fluorination.^30^ Today, this field of research is largely limited to processes relying on electrophilic fluorination. A notable exception is the asymmetric nucleophilic oxidative fluorination of ketoesters and aminofluorination of alkenes reported by Shibata and co-workers.^31^ An additional remarkable case of metal free catalytic nucleophilic fluorination involves the natural fluorinase discovered by O'Hagan and co-workers, an enzyme capable of inducing S_N_2 substitution with fluoride in water, a property exploited for the ^18^F-labeling of small molecules and peptides under mild conditions; this enzyme has not been used in the context of asymmetric fluorination.^32^ Electrophilic ^18^F-fluorination remains a challenging process for radiochemists due to the narrow range of ^18^F^+^ sources available to date and the difficulties associated with their preparation.^33^ Nevertheless, ^18^F^+^ radiochemistry offers great opportunities in asymmetric ^18^F-fluorination. An early example of organocatalyzed asymmetric fluorination is the chiral amine mediated electrophilic α-fluorination of aldehydes, a reaction independently reported by four research groups in 2005.^34^ Translation of this reaction to radiofluorination posed multiple challenges, especially in terms of avoiding the low temperature conditions and long reaction times associated with organocatalysis. Since α-fluoroaldehydes are prone to decomposition and racemization, they are generally further derivatized prior to analysis. For application to radiosynthesis this two-step procedure should ideally occur in one-pot, without time-consuming purification of the intermediate. Our laboratory recently reported that prochiral aldehyde substrates are amenable to asymmetric ^18^F-fluorination upon treatment in MTBE with stoichiometric chiral imidazolidinone (*S*)-I and [^18^F]NFSI,^35^ an ^18^F^+^ reagent synthesized from post-target produced [^18^F]F_2_.^36^ Notably, [^18^F]Selectfluor *bis*(triflate)^37^ was not a suitable ^18^F^+^ source for this transformation. After stirring for 20 minutes at room temperature, reagents for derivatization of the enantioenriched ^18^F-labeled aldehydes intermediates were added directly. The use of benzhydrazide in methanol afforded the corresponding hydrazone products with good radiochemical conversion (RCC) from [^18^F]NFSI and ee of up to 92% in a one-pot procedure (Scheme 5).

**Scheme 6:**
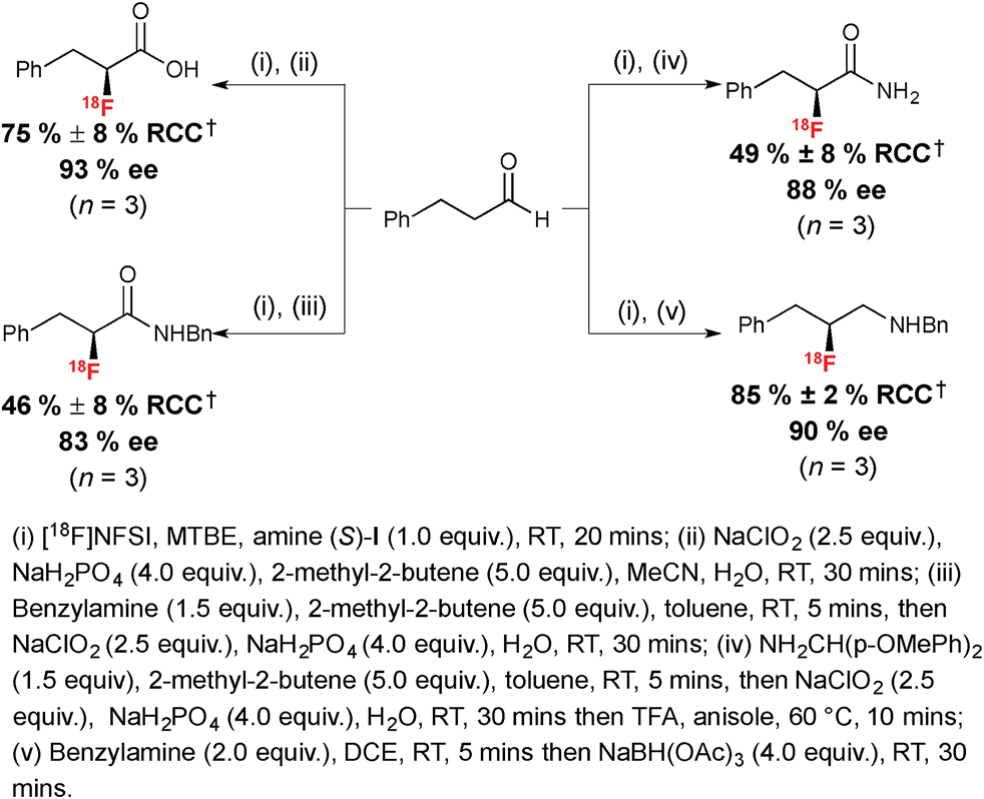
Radiosynthesis of enantioenriched ^18^F-labeled carboxylic acid, amides and amine. ^†^RCC determined by radio-HPLC relative to [^18^F]NFSI.

The utility of α-[^18^F]fluoroaldehyde synthons was further demonstrated with the preparation of an enantioenriched α-[^18^F]fluoro carboxylic acid, primary and secondary amides and a secondary amine product (Scheme 6).

The Pinnick–Lindgren oxidation performed with sodium hypochlorite in acetonitrile in the presence of sodium dihydrogen phosphate and 2-methyl-2-butene led to the desired ^18^F-labeled carboxylic acid with no erosion of ee. Oxidative amidation was performed in one pot; this process involved formation of an imine in the first instance, followed by a Pinnick–Lindgren oxidation affording the desired ^18^F-labeled amide with a slightly eroded ee of 83%. A representative enantioenriched ^18^F-labeled primary α-fluoroamide was also within reach in 49% RCC and 88% ee. Finally, a chiral β-fluoroamine was prepared from the enantioenriched aldehyde *via* imine formation in dichloroethane (DCE) followed by reduction with sodium triacetoxyborohydride.

**Scheme 7:**
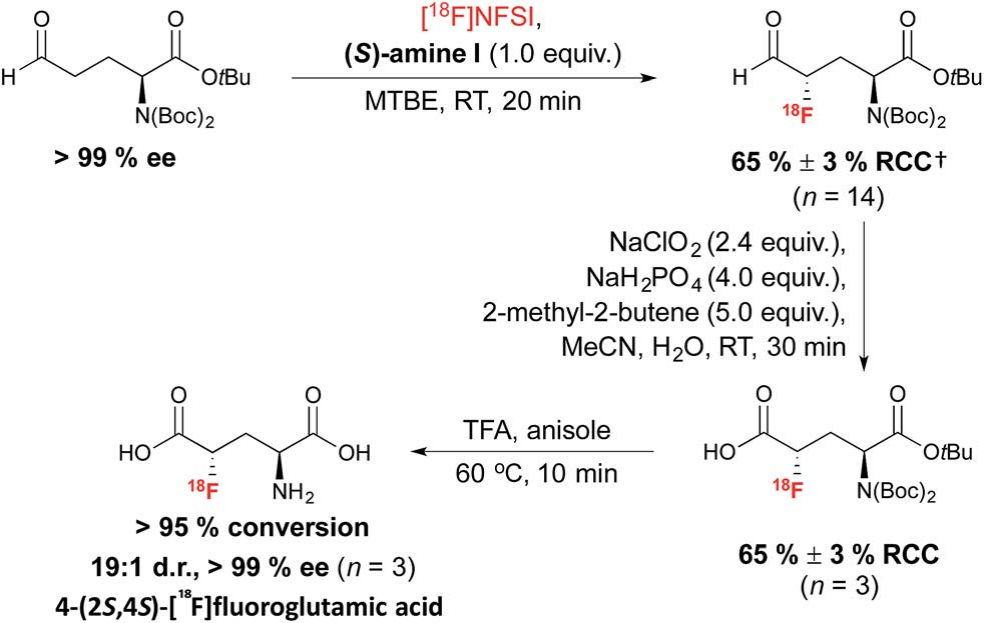
Organomediated radiosynthesis of [^18^F](2*S*,4*S*)-4-F-GLU. ^†^RCC determined by radio-HPLC relative to [^18^F]NFSI.

The method was subsequently applied to access (2*S*,4*S*)-4-[^18^F]fluoroglutamic acid (Scheme 7). The ^18^F-labeling of an enantiopure aldehyde precursor derived from l-glutamic acid under the optimized conditions, followed by a Pinnick–Lindgren type oxidation and TFA deprotection, afforded 4-(2*S*,4*S*)[^18^F]fluoroglutamic acid with very good RCC and d.r.; unlike the S_N_2 approach described in Scheme 2, this method did not lead to unwanted epimerization at C-2.^38^ Further experiments demonstrated a match/mismatch effect between the chiral imidazolidinone of opposite absolute configuration and the aldehyde substrate; therefore with (*R*)-amine I, significant erosion of d.r. was observed.

## 3. Conclusion

**Fig. 2:**
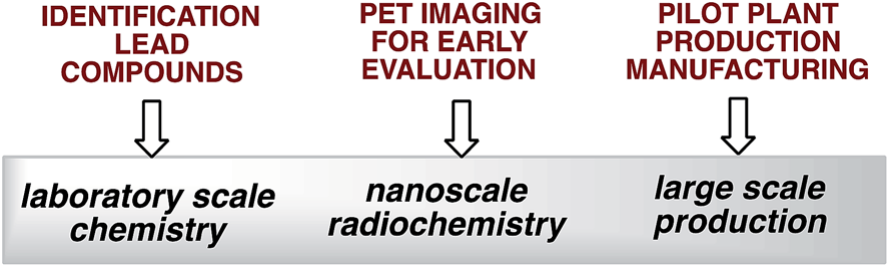
Synthetic scales in drug development.

There is ample evidence in the literature that PET imaging can facilitate the process of drug discovery and development. However, from a pragmatic viewpoint, the routine use of this imaging technology imposes non-trivial radiosynthetic challenges for medicinal chemists, especially when the drug candidate under study is a chiral non-racemic entity. Classical laboratory scale synthesis must be entirely revisited to allow for the nanoscale nature and challenges characteristic of ^18^F-radiochemistry necessary to evaluate potential drug candidates in a living system. Following these imaging studies and after passing all the hurdles of preclinical and clinical evaluation, large-scale production must then be implemented for the chiral non-racemic drugs selected for manufacturing operations (Fig. 2).

Such difficulties would be particularly stringent for chiral molecules with a stereogenic fluorinated carbon. These considerations pose a fundamental question on the real value of asymmetric ^18^F-fluorination for radiotracer production since one could argue that separation techniques of stereoisomers may be more rapid or cost effective; this is assuming that the identification of suitable separation conditions is fast and facile. A related debate arose with the realization that although asymmetric catalysis is an attractive method to control stereochemistry into pharmaceutically active molecules, in practice, other techniques are often used for pilot plant or production operations.^39^ This dichotomy results from industrial constraints for the application of asymmetric catalysis to the large-scale synthesis of drug candidates and commercial drugs. Such constraints include economic considerations, the speed to implement a particular process, the freedom to operate and process robustness. Today, most medicinal chemists would accept that the enormous progress made in asymmetric catalysis will be used by the pharmaceutical industry on a large scale when the catalysts employed are inexpensive, readily available and can be implemented rapidly. Similarly, it is likely that the development of a range of versatile and effective asymmetric ^18^F-fluorination reactions will be considered by radiochemists in the future as an alternative to separation techniques or conventional radiochemistry requiring multistep synthesis to access the chiral enantiopure precursors necessary for ^18^F-fluorination. To reach such a situation, it is important that our community continues to develop methods for effective asymmetric ^18^F-fluorination, employing readily available starting materials and mild reaction conditions, with a protocol that is easy to implement and ideally amenable to automation. The field of stereoselective and asymmetric labeling has been amply developed for a range of isotopes such as ^11^C, ^2^H or ^3^H.^40^ These advances have furthered our understanding of important biochemical processes and have highlighted the differential behavior of enantiomers in living systems. The radioisotope ^18^F is now receiving similar attention; this is an encouraging trend that, at a more fundamental level, should increase our understanding of the effect of chirality and F–C stereogenicity on living systems. New asymmetric catalytic fluorinations using transition metals, organocatalysts or other catalytic manifolds are being continuously developed in laboratories around the world; many of these methods offer attractive scope and selectivities. These will be used by radiochemists when the field of asymmetric ^18^F-fluorination has matured to a point where it is demonstrated that such strategy presents clear advantages over more conventional radiochemistry or separation techniques for rapid *in vivo* evaluation.
